# Editorial: Frontiers in psychodynamic neuroscience

**DOI:** 10.3389/fnhum.2023.1170480

**Published:** 2023-03-22

**Authors:** Filippo Cieri, Robin Lester Carhart-Harris, Christoph Mathys, Oliver Turnbull, Mark Solms

**Affiliations:** ^1^Department of Neurology, Cleveland Clinic Lou Ruvo Center for Brain Health, Las Vegas, NV, United States; ^2^Neuroscape, Weill Institute for Neurosciences, University of California, San Francisco, San Francisco, CA, United States; ^3^Centre for Psychedelic Research, Department of Brain Sciences, Imperial College London, London, United Kingdom; ^4^Interacting Minds Centre, Aarhus University, Aarhus, Denmark; ^5^Translational Neuromodeling Unit (TNU), Institute for Biomedical Engineering, University of Zurich and ETH Zurich, Zurich, Switzerland; ^6^School of Psychology, College of Human Sciences, Prifysgol Bangor University, Bangor, United Kingdom; ^7^Neuroscience Institute, The University of Cape Town, Cape Town, South Africa

**Keywords:** psychodynamic, psychodynamic neuroscience, cognitive neuroscience, psychoanalysis, neuropsychoanalysis, default network DN, neural networks, Freud

## Introduction

Parts of cognitive neuroscience still avoid the individual-subjective factor from their investigations, thereby excluding the brain's most unique feature, its selfhood. Many researchers in this field remain skeptical about the psychoanalytic approach and theories. Likewise, many psychoanalysts continue to eschew the structure and functions of the brain in their conceptions of the mind in physiological and pathological conditions. Both cases seem to preserve a Cartesian approach in which the mind is linked to the brain in some arcane manner, detaching subjectivity from the body—rather than treating it an integral part of the complex and dynamic organism as a whole. This approach gives rise on the one hand to a *mindless neuroscience*, and, on the other hand, to a *brainless psychoanalysis* (Cera et al.; Cieri). This Research Topic is an attempt to adopt a psychodynamic approach to cognitive neuroscience, and to furnish a natural science of psychoanalysis (Freud et al., 1954; Solms, 2020).

## Chapter 1: Hypotheses and theories

Since the renewed interest in the dialogue between neuroscience and psychoanalysis, especially after the advent of neuropsychoanalysis and studies on the Default Network (DN), Cieri proposes a philosophical and scientific proximity between time and self, suggesting a neural overlap in the DN. The author presents studies in cognitive neurosciences and functional Magnetic Resonance Imaging (rs-fMRI) to support his hypothesis. The ontogenetic development of self and time perception is discussed, consistent with the development of the DN's function. Alzheimer's disease is proposed as an example where perception of time is brutally impaired together with a loss of the self's functions.

Rabeyron is interested in a dialogue between psychodynamic therapy and the Free Energy Principle (FEP). Analytically oriented psychotherapy requires a setting, a particular mental state, and specific processes, to induce psychic transformations that can be seen in the light of the FEP. The paper supports a dialogue between psychoanalysis and the neuroscience of subjectivity.

Following a similar idea, Sikora is interested in the link between the theory of drives and the Fristonian FEP, with its hierarchical model of the brain, as a helpful integration of the Freudian economic approach. The author uses two psychodynamic traditions: the French and the post-Kleinian school of British psychoanalysis, as one side of the dialogue, with the FEP and the hierarchical predictive model on the other.

In their contribution, Scalabrini et al. focus on the nested hierarchy of self and its trauma, suggesting synchrony as a key factor in the processes of dialogue between self and others, which shapes the brain–body–mind system of the subject, including their sense of self. The authors use both Northoff's conceptualization of the brain-based nested three-layer hierarchical structure, and the three levels of trauma theorized by Mucci.

With a more philosophical approach, Brakel investigates the mind/body problem through a philosophy of mind framework, introducing issues for dualists and physicalists, along with key concepts such as independent mental causation, emergence, and multiple realization. To respond to some of these problems in a new light, this manuscript proposes a new mind/body approach: the Diachronic Conjunctive Token Physicalism (DiCoToP).

More interested in the psychotherapeutic technique, Chamberlin introduces the Active Inference Model of Coherence Therapy, based on Psychological Constructivism. This form of therapy suggests Coherence Therapy as a dyadic act of therapist guided Active Inference which renders conscious the potential unconscious causes of a patient's behavior. The author proposes this approach as a computational process useful for therapeutic help and experimental research design.

## Chapter 2: Original research

In their work, Fuchshuber et al. are interested in developing a standardized self-rated evaluation for the LUST emotion. The authors produce two versions of the L-scales (L-12 and L-5). Cronbach's α indicates good internal consistency regarding L-12 (α = 0.90), and acceptable value for the L-5 (α = 0.82). These scales demonstrate satisfying psychometric properties.

Quevedo et al. propose an experimental approach to explore a different field: the gene-environment interaction in the development of Borderline Personality Disorder (BPD). The authors use an epigenetic approach to show how molecular machinery adapts to the environment. They use a pilot study, with a small sample of BPD patients, exploring changes in peripheral DNA methylation of the FKBP5 gene, which encodes for a stress response protein, in relation to psychotherapy (both symptomatology and underlying psychological processes).

Bazan et al. have contributed to this Research Topic with two separate works. In the first, Olyff and Bazan recruited 1,458 participants using a rebus priming paradigm, where the images were followed by a target word semantically related to the rebus resolution, upon which the participants, unaware of the rebus principle, produced six written associations. The authors show how the images induced inadvertent rebus priming in naïve participants, suggesting that people solve rebuses unwittingly and independently of stimulus order, thereby constituting empirical evidence for the mental effectiveness of the signifier.

In their second experimental contribution, Thieffry et al. underline how the defenses measured in their research are internal, intimate control systems, probing for the censorship between the systems Unconscious and Preconscious. This study contributes support for a psychodynamic explanatory model of the production of parapraxes.

Schalkwijk et al. focus their research on Adverse Childhood Experiences (ACE's), potentially indexing vulnerability to maladaptive coping and stress, associated with insomnia. The authors used existing data from subjects with insomnia and normal controls, asking participants to complete the questionnaire about traumatic experiences during childhood, to explore the association between ACE's, shame coping-styles, adult insomnia, hyperarousal, and the neurobiology of autobiographical memory. Their findings can have implications for the treatment of insomnia, with more focus on traumatic experience and emotional processing rather than the typical (more superficial) sleep interventions.

Tanzilli et al. indicate that therapists highlighted different patterns of criticized/devalued and sexualized reactions to visual images of patients with distinct personality disorders, at statistically systematic and clinically meaningful levels. Moreover, psychotherapists' late positive potentials (LPPs) in the hippocampus were able to determine which patient they observed during the EEG task, with high accuracy.

Still in the field of the psychoanalytic setting and the investigation of brain correlates, Buchheim et al. explored change in the electroencephalographic (EEG) signal as an effect of psychoanalytical therapeutic interventions, investigating brain correlates of specific psychodynamic approaches in the EEG power spectrum. The authors contrast three types of intervention (clarification, confrontation, and interpretation) and a neutral control condition during a structured psychoanalytic interview conducted while the EEG was recorded. The authors were able to show that incisive interventions, such as confrontation with discrepancies and interpretation of unconscious intrapsychic conflicts, can stimulate temporary emotional lability, causing a change in psychic processing akin to interference from external stimuli.

## Chapter 3: Review, systematic review, and meta-analysis

Cera et al. use the approach of systematic review and meta-analysis to examine neural changes after psychotherapy, in several different mental disorders. The authors also attempt an exploration of the different psychotherapies' approaches, particularly interested in the comparison between psychodynamic and non-psychodynamic approaches. The meta-analysis and systematic review found that all psychological interventions influence the brain from a functional point of view, showing their effects from a neurological perspective. Frontal and prefrontal regions, insular cortex, superior and inferior frontal gyrus, and putamen are all involved in these changes following psychological therapy. Psychodynamic approaches are more prone to evoke changes in the latter three regions.

Williams and Trentini present an overview of the main contributions on intersubjectivity in the field of neuroscience. Based on the capacity for emotional resonance, a primary sense of connectedness is proposed by the authors, and potentially defined as intersubjective in that it entails shared affective states and intentions with caregivers. The authors propose to think of this kind of intersubjectivity as contingent. They also propose a multi-layered approach to intersubjectivity, consistent with current neuroscientific conceptualizations.

Tran The et al., are interested in the DN and how it can offer a useful field of investigation in the dialogue between mind and brain, particularly in the field of schizophrenia. Combining neuroimaging studies with Freudian hypotheses, the authors propose that hyperactivity of the DN is a consequence of a process of massive reassociation of traces in schizophrenia. This process may constitute an attempt at minimizing the excessive free energy that is present in psychosis.

Koslowski et al. try to connect the field of dream research, the predictive processing account of human cognition, affective neuroscience, neuropsychoanalysis, and emerging research on psychedelic substances, to deepen our understanding of the mechanisms of the dreaming brain and dream-like states. Conceptual bridges between theories of consciousness, dream research and neurobiological accounts are proposed, to further advance empirical studies on the nature of, and different functions of, dreaming.

## Conclusions

As editors, we are impressed by the quantity and quality of these contributions, often with an empirical—rather than only a speculative—approach to the problems proposed by this Research Topic. The psychodynamic approach in neuroscience—and dialogue between neuroscience and psychoanalysis more generally—is complex, and it holds a lot of open and challenging questions for future research. To disentangle this complexity, or at least to address it, the combined effort of multiple scientific approaches is needed—as underlined in this Research Topic. New approaches in formal education would also be useful, to build up a new generation of researchers, clinicians, and scientists, equipped with notions about the neuroscientific approach to psychoanalysis, and about the psychodynamic approach to cognitive neuroscience, recognizing—as mentioned—that subjectivity is an integral part of the complex and dynamic organism as a whole.

## Author contributions

FC: wrote the editorial. RC-H, CM, OT, and MS: read, corrected, commented, and approved the paper. All authors contributed to the article and approved the submitted version.
